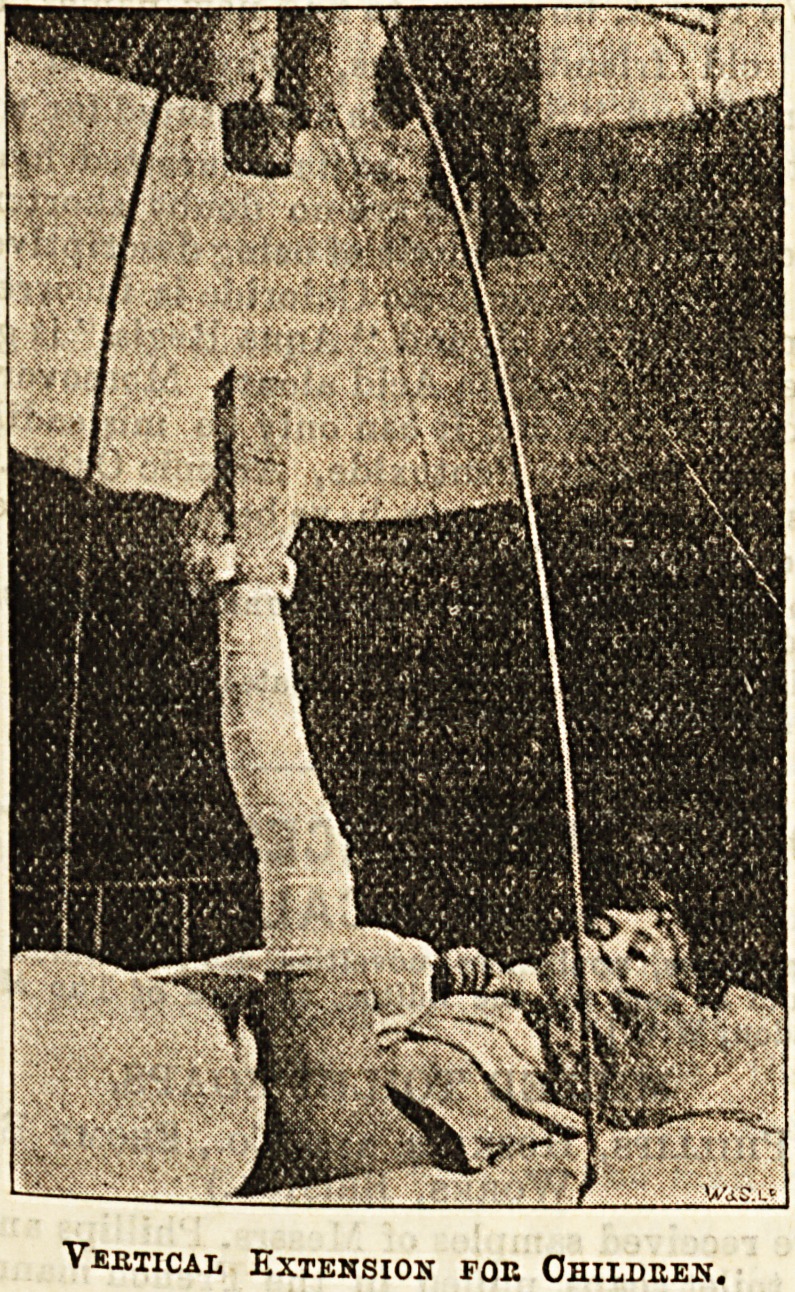# Treatment of Simple Fracture of the Femur

**Published:** 1893-04-01

**Authors:** 


### The Hospital Clinic.

[*The Editor will be glad to receive offers of co-operation and contributions from members of the profession All letters should be addressed to* The Editor, The Lodge, Porchester Square, London, W.]

### SUSSEX COUNTY HOSPITAL.

### Treatment of Simple Fracture of the Femur.

On admission into the hospital of a case of fractured femur the following routine preliminary treatment is adopted. The patient is removed from the surgery into the accident ward situated on the ground floor, and placed on a "fracture bed." The clothes are removed; the broken limb is extended by means of a folded towel wrapped round the ankle, and to which weights are attached by a string and hung over

Extension with Folded Towel.

the foot of the bed. Hot bottles are applied to the trunk and limbs, and restoratives administered internally. The patient is then washed, one limb only being exposed at a time, the remainder of the body being covered with blankets. Meantime the form of treatment is decided upon, and the splints and appliances prepared. The "fracture bed" is an iron bedstead 7 feet long, 40 inches broad, and 22 inches high. Deal boards, each 8 inches broad, are placed side by side close together across the bedstead, resting by their extremities upon the iron framework. These form a flat and unyielding surface for the matress. The boards are drilled with numerous holes one inch in diameter to allow evaporation from the matress, which without this device is very liable (even with great care) to become mouldy underneath. The matress is firm and made of horse hair. The foot of the bed is raised from the floor by wooden blocks, from four to six inches high, placed under the castors.

For the purpose of treatment simple fractures of the femur may be divided into—(1) fractures in and above the middle third; (2) fractures below the middle third; (3) fractures occurring in children.

Fractures in and above the middle third are usually treated as follows: The patient is anæsthetised, unless physical examination reveals any contra indication, chloroform being used by preference to promote complete muscular relaxation.

The apparatus in constant use may be briefly described:

1. Long outside splint, with a cross-piece near the lower extremity, of such a length that the upper end is in the axilla, and the lower about six inches below the heel. A large hole is made in the wood opposite the situation of the ankle to prevent pressure on the external malleolus. The splint is thickly padded.

2. Stirrup, composed of a flat piece of deal about three inches square, perforated in the centre of the flat surface, and surrounded on three sides by a strip of strong strapping about two inches and a half broad and five feet long, the adhesive surface facing the wood. A piece of knotted blind cord is threaded through the hole, with the knot inwards.

3. "Upright," consisting of a flat post, with a narrow slit running down the centre of the flat surface in the upper two-thirds of its extent. To this are attached by binding screws a pulley and a flattened hook, the former to transmit the extension cord, which is attached to the stirrup by one extremity, and to the weight by the other, the latter to attach the "upright" to the foot of the bed. Both pulley and hooks can be made to run freely up and down the slit, so that the "upright" can be readily adapted to any ordinary bedstead. A spike at the lower end, which is pressed into the floor, serves to steady the whole apparatus. A form of apparatus is sometimes used consisting of a metal framework with pulley attached. The ends of each link, as seen in the accompanying diagram, are forked and perforated with holes, so as to allow of their being fitted to the foot of the bedstead and screwed into any desired position. This form is not often used owing to the narrow range of its adjusting capacity, and also to the fact that the forked ends have a tendency to come apart and so interfere with the screws.

Upright; A, Front view; B, Side View, rigged.

4. Kettle-holder, or "Crimean splint," the latter being in more frequent use. This splint was recently reintroduced by Mr. Blaker, who considers it to be similar to that used by the Army Medical Department in the Crimean War. It is composed of thin strips of tinned iron about one inch broad, and eight or ten inches long, corrugated in their long axes to increase rigidity, and perforated near their edges in numerous places. Four or five of these are placed side by side close together on the back of a thin, flat, quilted pad of proportionate size, and sewn in that position. By this means a splint

Framework, with Pulley.

is produced, which is soft and easily adjusted, but at the same time possesses considerable rigidity in its long axis.

5. Body-band, consisting of a strip of canvas two yards long and a foot wide, used to bind round the thorax of the patient, and to include the upper part of the long splint. This is found to be much more convenient than bandages for the purpose, as being more comfortable, and less liable to ruck and shift its position.

6. Strips of strapping one inch wide, and of sufficient length to surround the leg at the calf with four inches to spare.

7. Small pads from four to six inches square for putting under the tendo achillis to raise the heel from the bed.

8. Bandages, sandbags, and weights.

When the patient is anæsthetised, and muscular relaxation complete, the foot on the injured side is firmly grasped by the nurse, one hand being placed under the heel and the other on the dorsum of the foot. Firm and steady traxion is then made, the foot at the same time being slightly raised from the bed. The house surgeon now applies the stirrup, laying one strip on each side of the leg, with the adhesive surface towards the skin. Each strip extends above the knee and below the heel. Sometimes these are applied directly to the skin, and sometimes a cotton bandage is put on first; the malleoli, however, are always protected by a piece of lint or a few turns of bandage. It is found that, unless the stirrup strips extend above the knee, effusion is apt to occur in that joint, especially if heavy weights are used. Strips of similar material, previously warmed on a strapping tin, or moistened with turpentine, are strapped over the strips above mentioned, round the limb in an oblique direction, each strip overlapping the preceding one. The strapping is begun about four inches above the ankle, so as to allow for a certain amount of slipping, which occurs sooner or later, and is carried up over the knee. The long outside splint is then attached to the outside of the limb by means of bandages. The end of a cotton roller bandage is fastened by pins to the padding of the splint just above the ankle, and the bandage given a few turns round the splint, then passed downwards and inwards under the limb, forming a kind of sling, which prevents the splint from shifting upwards. The bandaging is continued upwards as far as the limit of the strapping. A Crimean or kettle-holder splint is fastened with webbing straps over the site of fracture. Weights varying from five to twelve pounds are attached to the cord of the stirrup, which passes through the pulley. The body band is adjusted round the splint and thorax, on the same principle as that of the bandaging just mentioned. Finally, a soft pad is put under the tendo-achillis, and a sand-bag over the middle of the splint.

Every few days measurements of the limbs are taken and the weights altered, as occasion may require. The

Long Outside Splint Applied.

following method is usually adopted: The sound limb is placed in a position similar to that of the injured one, that is, with the knee straight and the foot pointed directly upwards. A mark is then made with an analine pencil over the most prominent point of the anterior superior spine on each side, and again over the apex of each internal malleolus. The distance between these two points is measured and compared, and the result noted.

2. Fractures occurring below the middle third of the femur are usually treated by means of the double inclined plane, with high side pieces, and extension applied to the whole splint by a cord with weights attached tied to the foot-piece, and carried over the foot of the bed. In measuring for this splint, the distance is taken from the flexure of the knee to the heel, and from the same point to the tuberosity of the ischium. The apex of the triangle formed by the double inclined plane, which must fit the flexure of the knee joint, is made the fixed point on the splint from which the measurements are taken in fitting it. The limb is simply placed on the splint, and the foot bandaged to the foot-piece. The side pieces, which are made twelve inches or more in depth, so that their lower edges may rest on the bed, are only padded along the upper part they are fastened in position by broad and strong webbing straps. When union is fairly firm, some form of support is applied to the thigh for a few weeks; this usually takes the form of a Thomas' splint, of which there are a considerable number in the hospital unused, and which

Double Inclined Plane.

can be readily adjusted to the patient's requirements by wrenches.

3. *Fractures of the femur occurring in children.*—If the child is old enough to obey, the long outside splint, with extension, is used, but a similar splint is usually put on the other leg also, to prevent rotation of the trunk. In children under four years the plan of slinging the fractured limb vertically by means of a stirrup and weights is frequently adopted. The stirrup is put

Vertical Extension for Children.

on with strapping, as in the adult, but instead of the pulley being fastened to a post at the foot of the bed, it is supported by an iron framework over the middle of the bed, so that the weights would, if heavy enough, lift the child from the bed. Sufficient weights are used to allow the surgeon's hand to be passed under the buttock without difficulty. The great advantage of this plan is that the child can easily be kept clean, and that steady extension is maintained on the broken limb, notwithstanding any amount of wriggling.

## Figures and Tables

**Figure f1:**
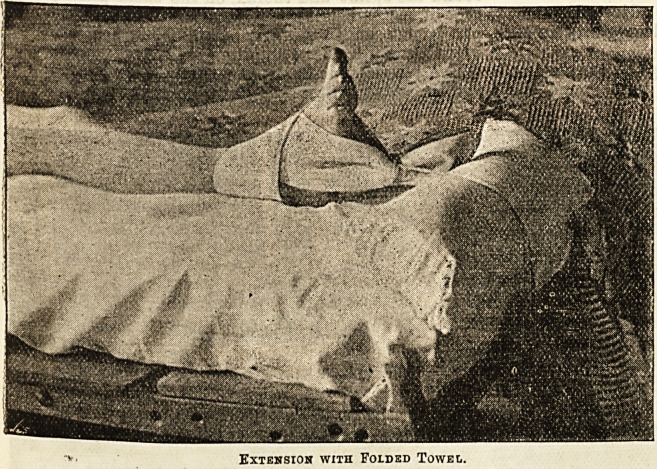


**Figure f2:**
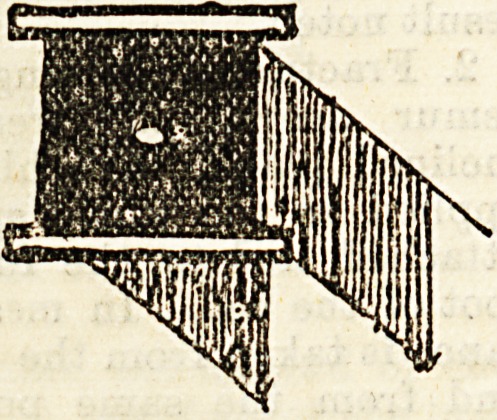


**Figure f3:**
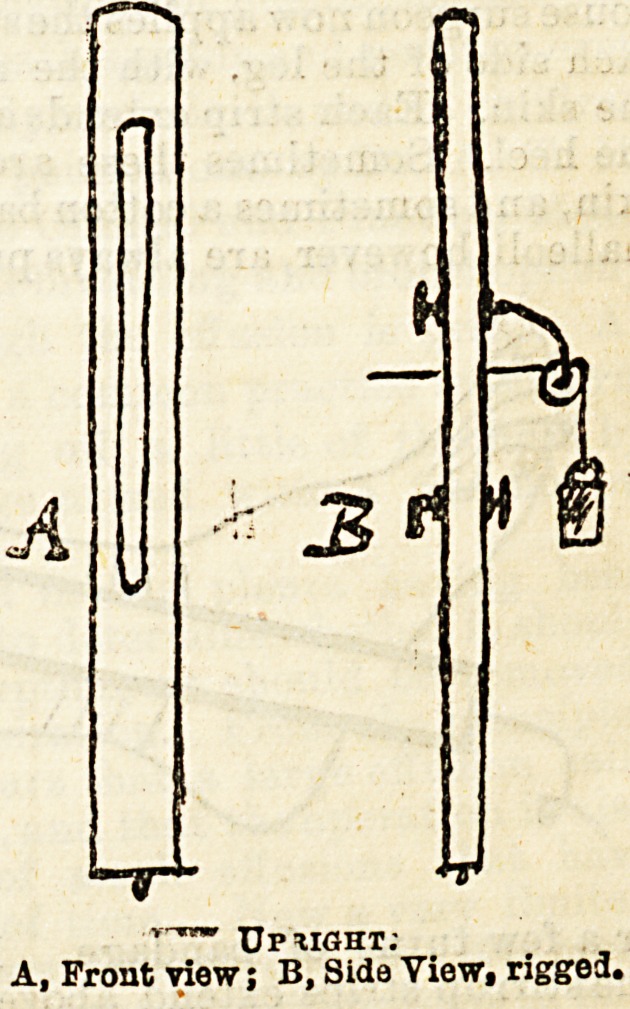


**Figure f4:**
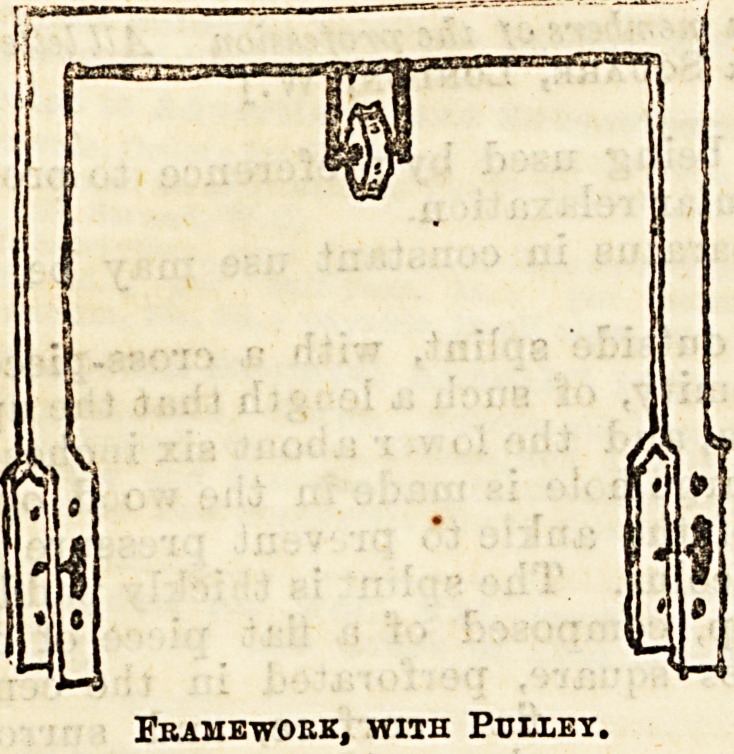


**Figure f5:**
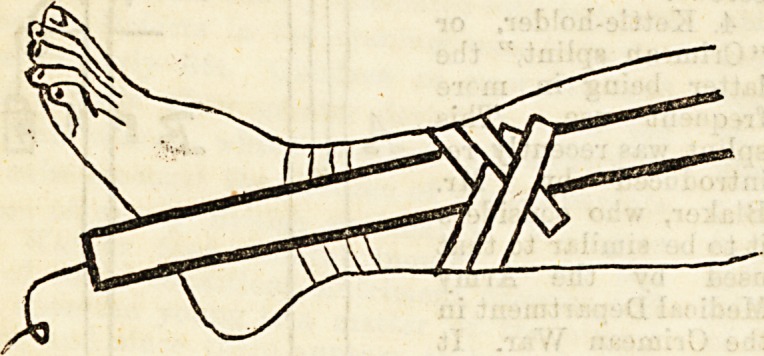


**Figure f6:**
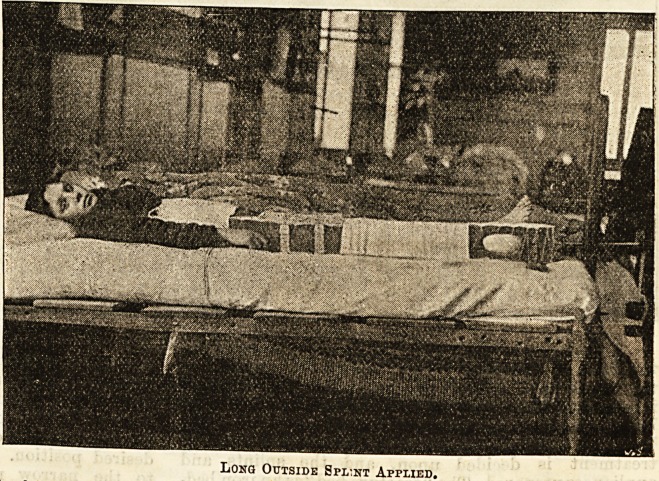


**Figure f7:**
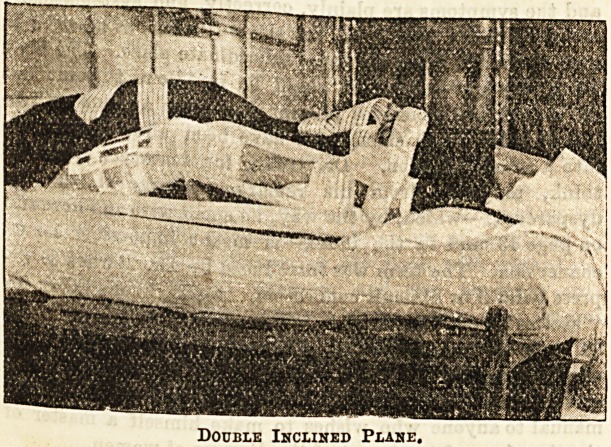


**Figure f8:**